# Chloroform Administration

**Published:** 1898-04-23

**Authors:** 


### Chloroform Administration.

Dr. Dudley Buxton not unnaturally feels that some apology is needed for addressing the world at large by way of the *Nineteenth Century* Review, on the subject of deaths under chloroform. Nevertheless, we may quite properly approve of the course he has taken, in that what he has done has given a very effective reply to the nonsense that the same paper had printed in a previous issue in praise of a reputed "safe method" of chloroform administration, a method which has been, perhaps more appropriately, described by a recent scientific observer as "the slap-dash method." Dr. Dudley Buxton gives to the Hyderabad Commission full praise for having demonstrated afresh the dangers to respiration which occur in administering chloroform, dangers which, as he says, have been recognised and carefully taught for over forty years. But he maintains that, when attempts are made to go beyond this, and to assert, as was asserted in a certain daily paper, that the Commission had shown that "to take chloroform was as safe as to take a tumbler of whiskey and water," it becomes the duty of those with technical knowledge to call a halt, and to point out that dangers do exist over and above that of an asphyxia caused by carelessness in administering chloroform. No death from chloroform due to asphyxia ought to happen, nor will it occur in competent hands in the case of an average patient. But there are other causes of death; and we think that Dr. Dudley Buxton is right in saying that "it is a severe and an unjust charge, unsupported by the weight of scientific evidence, to say that all deaths from chloroform are deaths from asphyxia, and are therefore unnecessary, avoidable, and consequently criminal deaths." We are quite sure that he is right also in saying that "it is distinctly a straining of truth to aver that those who have followed Syme's instructions . . . . have enjoyed an immunity from danger whilst giving chloroform." There can, we think, be no doubt that this slap-dash method is responsible for many of the deaths from chloroform, and we can hardly express the views which this journal has held on the subject better than in Dr. Dudley Buxton's own words: "All recognise the danger of paralysing the brain centres with chloroform, and so causing asphyxia, and always have recognised it, but the weight of both modern and less recent experiment and clinical observation compels us to recognise also the second and far more terrible danger, that of the onset of syncope from the direct action of chloroform upon the heart muscle."

